# Information Technology and Value-Based Healthcare Systems: A Strategy and Framework

**DOI:** 10.7759/cureus.53760

**Published:** 2024-02-07

**Authors:** Bakheet Aldosari

**Affiliations:** 1 Health Informatics, King Saud Bin Abdulaziz University for Health Sciences, King Abdullah International Medical Research Center, Riyadh, SAU

**Keywords:** it tools, clinician-patient interaction, patient care, value-based system, healthcare

## Abstract

Value-based healthcare offers a pathway for enhancing patient satisfaction and population health and reducing healthcare costs. In addition, it provides a means to enhance physicians’ perception and experience in healthcare delivery. The foundation of the said system is the notion that community wellness can only be benefited when the health effects of many people are also addressed. The provision of healthcare services incurs costs. However, a value-based model addresses this issue by establishing teams that cater to individuals with similar needs. This approach fosters expertise and efficiency, ultimately leading to cost savings without rationing. Furthermore, entrusting decision-making authority regarding healthcare delivery to the clinical team enhances doctors’ professionalism and the integrity of clinician-patient interactions, resulting in more effective and relevant treatments. Currently, various information technology (IT)-based solutions are the main focus for accomplishing the desired value-based healthcare system. The establishment of a coordinated framework that can help organizations create value-based healthcare systems is covered in the current article. Additionally listed are many IT-based solutions used to create a value-based healthcare system.

## Introduction and background

Value-based healthcare involves paying physicians and hospitals depending on their patients’ well-being. The related care agreements reward medical staff for helping patients make evidence-based health gains, reduce the occurrence and effects of chronic illnesses, and maintain healthier lives [1].

Value-based healthcare differs from capitated or fee-for-service models, where healthcare professionals are compensated based on the quantity of services rendered. In value-based healthcare, the focus shifts to evaluating the worth of healthcare by comparing the cost of achieving outcomes with the quality of those outcomes. This approach prioritizes the effectiveness and efficiency of care delivery over simply the volume of services provided. In broad terms, patients, medical professionals, consumers, suppliers, and the community all benefit from a value-based healthcare system.

The various advantages of the system mentioned above are as follows: First, patients spend fewer funds on enhancing their health. Managing chronic medical can be costly and time-intensive for individuals. The associated models prioritize accelerating patient recovery from illnesses and traumas and preventing chronic disease before it develops. Therefore, patients’ short- and long-term health evolves, requiring fewer doctor visits, tests, and treatments in addition to lower-cost prescription medicine.

Second, satisfaction among patients rises as care efficiency improves. Physicians may spend more time on new patient programs emphasizing prevention but less time addressing chronic illnesses when value precedes volume, quality, and patient engagement indicators increase.

Third, a healthy population makes fewer claims, reducing the pressure on payers’ investments and payment pools.

Fourth, vendors adjust prices based on patient outcomes. Manufacturers are being asked to link medicine costs to their actual patient benefit in light of the rise of personalized therapy. Numerous companies in the healthcare industry are engaged in such processes.

Fifth, the expense of healthcare decreases as populations get healthier. Value-based care has an opportunity to significantly reduce overall healthcare costs in a country where expenditure on healthcare makes up about 18% of the gross domestic product (GDP) [2].

The present article discusses the design of a coordinated framework that can guide organizations in building value-based healthcare systems. Various IT-based tools employed in building value-based healthcare systems are also enumerated.

## Review

An implementation framework for value-based healthcare

Value in healthcare is defined as the ratio of the cost of accomplishing a shift in a patient’s health consequences to that improvement itself [3]. Value-based care has been adopted to enable the healthcare system to provide patients with more value. It promotes professionalism, assists doctors in understanding their roles as healers, and is expected to be an effective arsenal in the battle against burnout. Measurable medical results demonstrate the capacity of physicians to collaborate with patients and their families to achieve outcomes, which helps to enhance the outcomes that matter most to patients and caregivers.

This innate desire is usually absent in the healthcare system, where professionals are routinely asked to put in long hours on tasks that do not significantly affect the health of their patients. Better outcomes also result in cheaper expenditures and lesser demand for ongoing care. By enhancing the well-being of patients, value-based healthcare reduces the progression of diseases and accumulative complexity, which raises the requirement for more treatment [4].

Organizations can be guided in developing value-based healthcare systems through an integrated framework. The following elements could be the stated framework’s main focus:

Understanding Patient Needs

Healthcare must be organized around clusters of patients with related medical requirements to be effective and productive. Clinical teams can effectively anticipate common patient needs and deliver frequently required services through a comparable care organization.

Design a Comprehensive Solution to Improve Health Outcomes

When the main focus of care transitions from solely treating patients to addressing their needs, care teams can attend to both the clinical requirements and the nonclinical factors that, if neglected, could jeopardize patients’ well-being. By recognizing and removing any gaps or roadblocks that compromise patients’ health outcomes, expanding and consolidating services leads to better outcomes [5].

Building a Team to Attain Solutions to Surfacing Patient Needs

When a team combines services, the necessity for coordinators diminishes, and in some cases, they may no longer be required. Informal conversations have significant potential to complement formal communication channels, ensuring the delivery of high-quality care, especially when team members are physically located in the same place. Moreover, this collaborative approach can be expanded beyond geographical boundaries, granting remote practitioners access to the latest knowledge and providing excellent healthcare locally, thereby eliminating the need for patients to travel [6].

Determining Health Outcome Efficiency

The information required to enhance care and effectiveness is also provided by evaluating health outcomes. Even though they must report reams of data, caregivers rarely track the health outcomes that are most important to patients and, consequently, to themselves as doctors. Moreover, the availability of cost and health consequences data facilitates condition-based bundled reimbursement models, empowering groups of caregivers to apply clinical judgment and regain their professional autonomy-two essential elements that contribute to job satisfaction and effective approaches to combat burnout [7].

Building Partnership

By prioritizing patients with similar needs and showcasing enhanced value in care, opportunities arise to foster collaborations and enhance wellness for a larger number of individuals. Employers are more prepared to work alongside healthcare providers and even contribute more per sector of care than they formerly would have been inclined to do, given that a quicker and more thorough recovery saves other employer expenses, such as those connected to absenteeism. For example, there is evidence of treatment with minimal adverse effects and permits patients to return to work quicker.

As healthcare teams gain expertise and develop the capacity to collaborate across various stages of the care cycle and multiple locations, connections among healthcare organizations are likely to grow. Integrated groups may engage with partners for several reasons, such as promoting lifestyle changes within a community, assisting rural clinicians in providing therapy to patients close to their homes, or leveraging advanced technology to exchange information with patients (Table 1).

**Table 1:** Major elements of IT system in building value-based healthcare system. TDABC: time-drive activity-based costing, VBHC: value-based healthcare, KPI: key performance indicator

Strategies of the IT system to approach VBHC		Summary
1. Patient-centric approach (consumerism)		Monitors patients across the whole continuum of treatment, including inpatient care at several sites, outpatient care, and all therapies. Instead of hospital, location, or department, data are summarized by the patient.
2. Comprehensive and coordinated care		Primary care physicians initiate and oversee patient treatment. When a patient sees multiple providers, such as a primary care physician and one or more specialization providers, that patient's care must be synchronized between those providers to guarantee that the care offered by all is efficient and effective. Value-based care coordination encompasses suitable communication and sharing of medical data.
3. Data-oriented strategies	Employment of common data definitions	Synchronization of all data fields, including diagnosis, labs, measures, and hospitalizations. Standardization of metrics, measurements, and data gathering techniques to enable data interchange throughout the entire system.
	Includes all patient data categories	Combines all data kinds (such as notes, pictures, prescriptions, and lab orders) for each patient. Keeps all data in one place.
	Enables accessibility and communication between all parties involved in providing care	Available to referring physicians and patients; it facilitates data sharing and aggregation among the various provider groups associated with each patient.
	Incorporating models and expert systems for every medical problem	Offers medical condition perspectives and templates to improve data entry, search performance, and input of standard order sets. Integrates expert systems to enhance decision-making, evidence-based care, and the follow-up of clinical alarms (such as test results, drug interactions, allergies, and others).
	Easy extraction and report information	Capability to accurately assess and record patient-specific outcomes, process metrics, expenses (like TDABC), and health risk factors (like comorbidities). Uses natural language processing and structured data sets (rather than free text) to reduce the requirement for manual data abstraction and the recruitment of personnel with expertise in data abstraction. Integrates outcome and cost metrics into the meaningful use requirements for electronic medical records. Automatically incorporates patient-specific transaction durations using time-stamped data when available; streamlines public reporting of results and expenses.
4. Organizational operation: efficiency	KPIs and dashboards; revenue cycle management	We can gauge the value of healthcare by using key performance indicators (KPIs). As we proceed to move away from a volume-based system and toward a value-based approach for reimbursement and public reporting, these measures are crucial in the present healthcare business landscape. The basics of managing the enormous volumes of data generated by every healthcare firm include creating a fantastic dashboard, improving analytics and reporting for your team, and picking the appropriate KPIs.
5. Regulatory and third-party payers	Governmental regulations; public health; reimbursement	Governmental organizations can enact regulations that encourage service providers to adhere to a set of quality, equitable, and cost-effective care requirements. In order to participate in structured programs, organizations may demand that healthcare providers abide by the quality and safety criteria established by certain third parties.

Various IT-based applications employed in building value-based healthcare systems

Electronic Health Records (EHRs)

The usage of electronic health records (EHRs) is becoming progressively prevalent. According to medical professionals, EHRs were originally expected to make value-based care more easily implemented. An EHR keeps track of a patient’s medical and administrative conversations with a provider throughout patient care sessions. As a result, the EMR reflects the work habits, knowledge, and skills of the healthcare professionals who developed it. It inadvertently includes patterns of data and elements that describe the systems of these suppliers.

Utilizing technology is primarily done so that we may easily access all the data we require for patient care, education, and practice administration at the moment of care [8].

*Computerized Physician Order Entry​​​*​​​​* (CPOE): E-prescribing*

Computerized physician order entry (CPOE) is inputting medical orders, including drug prescriptions, using a computer or smartphone application. Initially, the main purpose of CPOE systems was to simplify the online ordering of examinations, therapies, consultations, and prescription drugs [9]. These systems are often coupled with clinical decision support systems (CDS), which serve as tools to reduce errors by providing prescribers with information on recommended drug doses, administration routes, and intervals for administration [10].

Electronic Sign-Out and Hand-Off

It entails the “temporary or ongoing transfer of professional duty and responsibility for certain or every element of care for a patient, or set of patients, to a different individual or professional group” [11]. Enhanced work-hour limitations in certain residency programs, which reduced the consistency of care and enhanced the frequency of alterations to shifts, made the transition of professional responsibility more noticeable to residents [12]. Uncertainty over the transfer of unit accountability increased due to the segmentation of healthcare due to the growth of sub-specialties, which increased the number of transitions and hand-offs for a single patient [13]. Given that they involve a significant level of risk, hand-offs are an objective for quality upgrades.

Clinical Decision Support (CDS)

Clinical decision support (CDS) offers healthcare professionals relevant facts and patient-specific data to aid them in making well-informed and evidence-based decisions regarding patient care. The data are carefully filtered and appropriately presented to the healthcare professional to influence their decision. In a typical CDS system (CDSS), patient information is entered into a computerized clinical knowledge base, generating patient-specific evaluations or recommendations for the physician to consider. This program will provide clinical decision-makers instant support [14].

The effectiveness of various CDSS modifications to improve physicians’ adherence to alerts was investigated in several clinical trials. It was found that “tiering” and “automation of alerts” improved doctors’ compliance with CDS notifications [15,16].

Patient Data Management Systems (PDMSs)

Health data management underwent disruptive shifts with the transition of medical records from paper charts to EHRs to offer more precise and superior patient treatment while using this information qualitatively. Various health data management system concepts emerged due to the development of ITs supporting this transformation. Especially because of the increased frequency of data breaches and cyberattacks, security and privacy are the main requirements for a healthcare data management system [17]. Big data analytics requires hospitals to share patient information to obtain findings and predictive analysis from the data. It opens the door to a health data management system that will assist doctors and other medical professionals in improving chronic disease diagnosis and prognosis [18]. 

Patient Electronic Portals and Mobile Application

Patient portals, hospitals, and other healthcare providers can make obtaining their EMR data easier [19]. Griffin et al. (2016) stated that patient portals can offer reliable, online accessibility to personalized health information [20,21].

Telemedicine

Telemedicine uses telecommunications technologies to improve contact between patients and doctors or between doctors and patients. Real-time, two-way video communication enables synchronous and asynchronous patient clinical data transfer. Apart from communication, telemedicine allows for remote health data collection from medical equipment or personal mobile devices. This data can track, monitor, or modify patient behavior in the healthcare management process [22,23].

Electronic Incident Reporting, Patient Safety, and Medical Errors

The reporting systems allow safety experts to study occurrences, find underlying causes, and produce beneficial information to reduce risks by gathering adverse events and near misses in the healthcare industry [24]. The gathering and evaluation of events seem to be done more quickly than with conventional paper-based methods since the introduction of electronic patient safety reporting (e-reporting) systems [25]. Previous research has shown that user interface-related human factors can significantly impact the quality and pace of event reporting [26]. A well-constructed electronic reporting system could act as a catalyst for improving data quality for patient safety and lowering medical mistakes.

Revenue Cycle Management

Healthcare facilities utilize medical billing software to oversee patient care sessions, starting from registration and appointment scheduling to the eventual settlement of outstanding balances. This comprehensive process is referred to as revenue cycle management (RCM). When integrated with other health IT platforms, an efficient RCM system can significantly reduce the time between service delivery and payment collection as patients receive treatment [27].

Dashboards

These are business intelligence tools for gathering, processing, and displaying performance measure data. Dashboards should enable users to easily display useful data that can be used to improve clinical and organizational effectiveness. In reality, dashboards are often integrated into large, complicated healthcare organizations with end users with specific needs [28]. An advanced healthcare analytics framework, a medical dashboard, collects data from various sources, provides a comprehensive assessment of the performance of indicators for the hospital as a whole team, and enables speedy data analysis and application. The main objectives of these technologies are to improve data reporting and analysis, eliminate blockages like bad or erroneous data, and provide pertinent information.

Public Health Informatics (PHI)

Public health informatics (PHI) is the deliberate implementation of information, computer science, and technology in public health. One of the primary uses of PHI is to advance community health, which will eventually improve individual health. Modifying the factors that put the population at higher risk of disease and injury to prevent these occurrences [29]. In essence, PHI employs informatics to collect, examine, and act on data related to public health. PHI differs from other informatics disciplines in that it places a high premium on minimizing illness in the community, uses various techniques to accomplish its objectives, and operates in governmental settings [30]. The purview of PHI includes the conception, development, implementation, deployment, amplification, upkeep, and monitoring of communication, surveillance, and information systems pertinent to public health [31,32].

AI-Enabled Healthcare Systems

AI and related technologies are currently beginning to be applied in the healthcare industry and are spreading throughout communities and sectors. Numerous investigations have already demonstrated that AI can carry out crucial healthcare tasks, such as disease detection, at a pace with or superior to human beings [33]. The Internet of Things (IoT) is widely used to connect readily available medical facilities and provide trustworthy and efficient intelligent healthcare [34]. A few IoT healthcare applications include glucose monitoring, body temperature evaluation, oxygen saturation evaluation, medicine management monitoring and detection, essential healthcare services, and many more [35-37].

In IoT-related healthcare systems, wearable devices are at the forefront of every discussion because they can contribute to and bring about a significant revolution. In the past two decades, researchers have developed a variety of wearable gadgets that can track important health parameters like temperature, pulse rate, respiration, blood pressure, lung sound, and breathing.

Incorporating Patient-Generated Health Data (PGHD) Into Healthcare Information Systems

PGHD are health-associated data gathered or recorded by patients to help them understand their health and self-care capacity. In contrast to other patient-reported outcome information, the collection of PGHD data is patient-driven rather than practice- or research-driven. Technical tools that help patients gather PGHD can be useful for illness prevention and management since they encourage self-management practices like regular exercise and a healthy diet.

## Conclusions

The transition from a conventional framework to one based on values will require time, and it appears to be more difficult than first thought. As the healthcare market develops and more value-based care models are adopted, providers may endure short-term financial losses while long-term costs decline. Even while the number of healthcare providers participating in value-based care initiatives is growing appreciably, many still do not favor the same. Future models in the public and commercial sectors, especially for those serving underserved or rural communities, would probably benefit from being more accessible and financially lucrative to boost participation.

More research is required into the effects of these programs on patients, providers, and the healthcare system as a whole, as well as the variables that predict success.
